# Linking Metacognition and Mindreading: Evidence From Autism and Dual-Task Investigations

**DOI:** 10.1037/xge0000878

**Published:** 2020-09-10

**Authors:** Toby Nicholson, David M. Williams, Sophie E. Lind, Catherine Grainger, Peter Carruthers

**Affiliations:** 1 School of Psychology, Keynes College, University of Kent; 2 Department of Psychology, City, University of London; 3 Department of Psychology, University of Stirling; 4 Department of Philosophy, University of Maryland

**Keywords:** autism spectrum disorder, metacognition, mindreading, dual-task, theory of mind

## Abstract

Questions of how we know our own and other minds, and whether metacognition and mindreading rely on the same processes, are longstanding in psychology and philosophy. In Experiment 1, children/adolescents with autism (who tend to show attenuated mindreading) showed significantly lower accuracy on an explicit metacognition task than neurotypical children/adolescents, but not on an allegedly metacognitive implicit one. In Experiment 2, neurotypical adults completed these tasks in a single-task condition or a dual-task condition that required concurrent completion of a secondary task that tapped mindreading. Metacognitive accuracy was significantly diminished by the dual-mindreading-task on the explicit task but not the implicit task. In Experiment 3, we included additional dual-tasks to rule out the possibility that *any* secondary task (regardless of whether it required mindreading) would diminish metacognitive accuracy. Finally, in both Experiments 1 and 2, metacognitive accuracy on the explicit task, but not the implicit task, was associated significantly with performance on a measure of mindreading ability. These results suggest that explicit metacognitive tasks (used frequently to measure metacognition in humans) share metarepresentational processing resources with mindreading, whereas implicit tasks (which are claimed by some comparative psychologists to measure metacognition in nonhuman animals) do not.

Questions of how we know our own minds, how we know other minds, and whether nonhuman animals are capable of such self- or other-awareness, have proven to be some of the most enduring and important in the history of psychology and philosophy. The experiments described here were designed to throw light on both sets of questions at once. We begin by discussing the relationship between self-knowledge and other-knowledge in humans.

*Metacognition* (metarepresentation of one’s own mental states) is considered essential for day-to-day behavioral functioning because it is this monitoring of one’s internal states that allows one to regulate those states (Nelson & Leonesio, 1988). *Mindreading* (metarepresentation of others’ mental states; sometimes referred to as “theory of mind”) is likewise important for almost all domains of human social life, and when it is diminished most aspects of social life suffer. For example, autism spectrum disorder (ASD) is a developmental disorder diagnosed on the basis of significant impairments in social-communication and behavioral flexibility (American Psychiatric Association, 2013) and is characterized unambiguously by diminished mindreading ability (e.g., Happé, 1995). Thus, the clinical significance of understanding the relationship between mindreading and metacognition is high.

A number of opposing theories have been proposed, offering differing accounts of the relation between these two important abilities. For example, some have thought that mindreading develops ontogenetically and phylogenetically from an existing metacognitive system along with additional imagination/mental simulation abilities (*metacognition-is-prior* theory; e.g., Goldman, 2006). Others have claimed that metacognition and mindreading rely on entirely distinct systems (*two-systems* theory; e.g., Nichols & Stich, 2003). And yet others have argued that both involve a single metarepresentational system that processes both the mental states of self and of others (*one-system* theory; e.g., Carruthers, 2009, 2011). Crucially each of these theoretical accounts provides differing predictions for how these abilities might relate to each other experimentally. In the current investigation, we tackled these issues using complementary approaches in three experiments.

Before getting to the experiments, however, something further should be said about what the talk of “systems” here amounts to. Consider the one-system view first. This holds that there is a single core competence—involving a common set of conceptual resources and implicating the same mindreading network in the brain—underlying both our attributions of mental states to other people and to ourselves. While many of the inputs to the system may differ across the two cases (e.g., including one’s own inner speech, bodily feelings, and visual imagery in the first-person case only), essentially the same cognitive and inferential resources are involved in processing those inputs. In the version proposed and defended by Carruthers (2011), moreover, the system evolved in the first instance for third-person social purposes. Self-awareness results from turning that third-person system on the self.

In contrast, the two-systems view maintains that there are distinct and dissociable networks in the brain responsible for attributing mental states to other people and to oneself, though it can be left open whether or not the two systems draw upon the same set of conceptual resources (Nichols & Stich, 2003). Hence one’s capacities to draw inferences about the mental states of others can be damaged or interfered with without damaging or interfering with one’s ability to attribute mental states to oneself, and vice versa. This view can remain neutral on the question of the evolutionary emergence of the two capacities.

Finally, the metacognition-is-prior account maintains that attributing mental states to other people implicates a prior ability to attribute those states to oneself. It is by first being aware of one’s own mental states, and then projecting such states to other people using one’s imaginative or simulative abilities, that one comes to understand the minds of others. On this view, there will be a common set of conceptual abilities involved in both self-attribution and other attribution, but the cognitive and inferential resources required for the latter will go well beyond what are needed for self-awareness (Goldman, 2006). A prediction of the view, then, is that mindreading abilities can be lost or interfered with without losing or interfering with metacognitive ones, but any task that interferes with the metacognitive component of mindreading will thereby interfere with the latter also. This view is often understood to go along with the claim that metacognitive abilities emerged in phylogeny prior to mindreading ones.

Turning now to our experiments, in Experiment 1 (case-control experiment), we tested metacognition (using a classic “judgement of confidence” paradigm) and mindreading in children and adolescents with ASD, as well as in age- and IQ-matched neurotypical (NT) comparison participants. We know that ASD tends to involve a mindreading impairment, so if the one-system theory is correct, then participants with ASD should also manifest difficulties with metacognition. In contrast, both metacognition-is-prior and two-systems theorists have predicted explicitly that metacognition will be unimpaired in ASD, claiming that autism will reveal a crucial dissociation between metacognition and mindreading that would support their theories (Goldman, 2006; Nichols & Stich, 2003; Raffman, 1999; Wojcik, Waterman, Lestié, Moulin, & Souchay, 2014).

As we have just noted, actual proponents of the metacognition-is-prior view have predicted that metacognition will be unimpaired in ASD. This is because they have thought that the impairment in mindreading in ASD is likely to result from the known difficulties such people have with imagining and pretending and because they think that mindreading results from a combination of metacognition with imagination or simulation (Goldman, 2006). But such a position isn’t mandatory for a metacognition-is-prior theorist. It could be maintained instead that the difficulties with mindreading in ASD result from an underlying metacognitive deficit. In that case, this view, too, would predict that metacognitive abilities will be impaired in autism. This form of metacognition-is-prior view is not directly tested in the experiments that follow. But notice that if we succeed in showing (both here and in Nicholson, Williams, Grainger, Lind, & Carruthers, 2019) that the tests of metacognition employed with monkeys are not genuinely metacognitive in nature, then one significant strand of support (as detailed below) for all metacognition-is-prior views will have been undermined. In what follows, when we refer to the metacognition-is-prior theory we intend just the version defended by Goldman (2006), which predicts intact metacognition in ASD.

In Experiment 2 (dual-task experiment), instead of studying metacognition in a population with existing mindreading difficulties we tried to, in a sense, induce mindreading difficulties in neurotypical people by using a dual-task paradigm. In a dual-task paradigm, participants complete a primary task either alone (single-task condition) or concurrently with a secondary task (dual-task condition). If the primary and secondary tasks share processing resources, then primary task performance will be significantly poorer in the dual-task condition than in the single-task condition, because both tasks are competing for a limited resource. In Experiment 2, neurotypical adults completed metacognition tasks either alone or alongside a secondary task that tapped mindreading. If metacognition depends on the same metarepresentational resources as mindreading, then metacognition should be significantly poorer in the dual-task condition. The two-systems view, at any rate, makes no such prediction.

We turn now to the question of whether nonhuman animals are aware of their own and other minds. It turns out that this is directly related to the question of whether metacognition depends on the same metarepresentational resources as mindreading. Some theorists have suggested that some species of nonhuman primates (macaques, in particular) are capable of metarepresenting their own epistemic mental states, despite apparently lacking the equivalent ability to metarepresent the epistemic mental states of conspecifics (Beran, Smith, Coutinho, Couchman, & Boomer, 2009; Martin & Santos, 2014; Smith, Beran, Couchman, & Coutinho, 2008; Smith, Couchman, & Beran, 2014; Son & Kornell, 2005). If this interpretation of the data is correct, then these findings challenge the one-system view but are in keeping with both other views.

However, some have argued that the tasks used to assess metacognition in nonhuman primates are not truly metacognitive (do not require metarepresentation of self), claiming that they can be successfully completed using first-order, nonmetacognitive processes rather than second-order, genuinely metacognitive processes (Carruthers, 2011). That is, it has been said that instead of monitoring their own states of certainty or uncertainty, as most comparative psychologists assume (a second-order, metacognitive, process), the animals can succeed in these tasks through first-order (cognitive rather than metacognitive) kinds of risk-evaluation (Carruthers & Ritchie, 2012).

In the current study, we set out to test these competing interpretations of the animal data, too. We were able to do so because if humans solve the sorts of task conducted with monkeys without employing metacognition, then this provides at least a “proof of concept” that the monkeys are doing so too. We therefore employed a version of the “gambling task” introduced by Son and Kornell (2005), which has been used to assess metacognition in nonhuman primates (Kornell, Son, & Terrace, 2007). This task is in many respects structurally equivalent to the classic judgment-of-confidence task used to measure metacognition in humans (and which we also use in the current study). Strictly speaking, however, the gambling task measures the degree to which strategic behavioral responses are accurate (rather than the extent to which verbal metacognitive judgments are accurate, as in the explicit judgment-of-confidence task). In what follows, we refer to the gambling task as “the implicit task” (because it is, putatively, implicitly metacognitive in nature) and the judgment-of-confidence task as “the explicit task” (because it requires participants to reflect explicitly on their own internal states and provide a verbal response).

In the task employed with monkeys by Kornell et al. (2007), the animals were first required to make a primary discrimination of some sort (e.g., judging the longest of a set of lines) before being asked to place a “bet” on the correctness of that choice. Having made their initial discrimination, they could either select a “high risk” symbol that would issue in a large reward if they had made the correct choice and a large loss if they had got it wrong, or they could select a “low risk” symbol that gave small payoffs or penalties. According to the authors, the monkeys solve this task by monitoring their uncertainty about the correctness of their initial choice, gambling or declining to gamble accordingly. The task is thus thought to demonstrate the presence of metacognitive processes in these animals.

An alternative, nonmetacognitive interpretation of the data is also possible, however. For we know that easy tasks are ones that are performed fluently, and fluency is known to give rise to positive valence, whereas disfluent tasks cause negative valence (Casasanto & Chrysikou, 2011; Carr, Rotteveel, & Winkielman, 2016). So a primary discrimination task that is executed fluently will result in a state of positive affect, which is known in humans to increase risk-taking (Gasper & Clore, 2000). Hence the high-risk option will seem good to participants following fluent performance. In contrast, disfluent execution will produce negative affect, making the high-risk option seem bad. Metarepresentation of self is thus unnecessary to produce accurate performance on the gambling task.

It is worth noticing that processing fluency is among the cues that humans frequently use when making explicit metacognitive judgments (Dunlosky & Metcalfe, 2009; Hertzog, Dunlosky, Robinson, & Kidder, 2003). But, of course, the fact that processing fluency can provide a cue for a metacognitive judgment doesn’t mean that processing fluency is itself metacognitive. Ease of processing is one thing; awareness that processing is easy is another. Only the latter is metacognitive in nature.

One of our goals was to discriminate between the two competing explanations of the cognitive resources employed in the implicit task (metacognitive or nonmetacognitive). If the implicit gambling tasks that have been used with monkeys really do require metarepresentation of self, then gambling accuracy should be diminished to the same extent as judgment-of-confidence accuracy in (a) participants with ASD relative to comparison participants in Experiment 1 and (b) the dual-task condition relative to the single-task condition in Experiment 2—provided, that is, that metacognition and mindreading share resources, as postulated by one-system views and metacognition-is-prior theories.

A complete set of predictions for both experiments is shown in Table 1 (our own predictions are listed in the bottom row). Note that our task design enabled us simultaneously to pit the one-system account of the relation between mindreading and metacognition against its opponents, as well as pitting first-order against metacognitive interpretations of the implicit gambling tasks that have been used with monkeys.[Table-anchor tbl1]

## Experiment 1

### Method

#### Participants

Twenty-five children/adolescents with ASD and 25 neurotypical (NT) comparison children/adolescents took part in the study. All participants completed the Wechsler Abbreviated Scale for Intelligence-II (Wechsler, 1999), which provides verbal, performance, and full-scale IQ scores, as well as two widely used measures of mindreading (see details below). Mindreading tasks were included to ensure the ASD group was representative of the population in showing a mindreading impairment (otherwise the predictions of one-system theory could not be tested in this experiment).

Participants in the ASD group had received verified diagnoses, according to conventional criteria (American Psychiatric Association, 2000; WHO, 1992). The parents of all participants completed the Social Responsiveness Scale-II (Constantino & Gruber, 2012) to provide an index of number of ASD features/traits manifested in participants (*T* scores of 50 represent the population average; *T* scores >59 indicate clinically significant levels of ASD traits/features). Participant characteristics and group matching statistics are presented in Table 2. All participants had normal or corrected-to-normal vision, and none of them was color blind. Ethical approval for Experiments 1 and 2 was given by the School of Psychology, University of Kent Research Ethics Committee (Ref: 201815281154425026), and informed consent was obtained from the parents of all participants and the participants themselves.[Table-anchor tbl2]

#### Materials and methods

##### Mindreading task 1

The Reading the Mind in the Eyes Task (RMIE; Baron-Cohen, Wheelwright, Hill, Raste, & Plumb, 2001) is a widely used measure of mindreading in clinical and nonclinical populations. Participants completed a child version of the RMIE task (Baron-Cohen, Wheelwright, Spong, Scahill, & Lawson, 2001). The child version includes 28 photographs of the eye-region of the face taken from the adult version of the task. On each trial, participants were asked to pick one word from a selection of four to indicate what the person in the picture was thinking or feeling. If participants felt more than one of the words was applicable, they were instructed to select the word they thought was most suitable. Stimuli were presented on screen to participants in a random order, and no time limit was imposed. Scores on the RMIE task range from zero to 28, with higher scores indicating better performance on the task. The proportion of items correctly identified by ASD and comparison participants is shown in Table 2.

It should be noted that although the RMIE can be characterized as a kind of empathy task, it is also undoubtedly a task that requires mindreading of the mental states of the target agents. For in each case what has to be selected is the most appropriate mental-state descriptor. The task has been employed in over 250 studies, shows good test–retest reliability (e.g., Fernández-Abascal, Cabello, Fernández-Berrocal, & Baron-Cohen, 2013), clearly distinguishes groups of participants with and without ASD (e.g., Wilson et al., 2014), is associated with the number of ASD traits shown by individuals in large population studies (e.g., Baron-Cohen et al., 2001), and is correlated with other measures of mindreading even after the influence of IQ is statistically controlled (e.g., Jones et al., 2018).

##### Mindreading task 2

We employed a version of the Animations Task (Abell, Happé, & Frith, 2000) as a second measure of mindreading. The task, which is based on Heider and Simmel (1944), required participants to describe interactions between a large red triangle and a small blue triangle, as portrayed in a series of silent video clips. Four such clips are apt to invoke an explanation of the triangles’ behavior in terms of epistemic mental states, such as belief, intention, and deception. These clips comprise the “mentalizing” condition of the task and were employed in this study.

Each clip was presented to participants on a computer screen. After the clip was finished, participants described what had happened in the clip. An audio recording of participants’ responses was made for later transcription. Each transcript was scored on a scale of zero to two for accuracy (including reference to specific mental states), based on the criteria outlined in Abell et al. (2000). Eighty percent of transcripts were also scored by two independent raters, each of whom was blind to the diagnostic group of the participant and any other results from the study. Interrater reliability was excellent according to Cicchetti’s (1994) criteria (intraclass correlations >.84). Accuracy (proportion) among ASD and comparison participants is shown in Table 2. Note that the Animations Task is a measure of spontaneous mental-state attribution. Those who score highly on this measure are spontaneously interpreting the movements of the geometric figures in the videos in mental-state terms. Those who score low on this measure, in contrast, will tend to provide literal (nonmentalistic) descriptions of the movements they have observed.

##### Experimental gambling and judgment-of-confidence tasks

There were two experimental tasks (implicit gambling and explicit judgment-of-confidence), both of which involved an object-level component that required participants to make perceptual discrimination judgments and a (supposed) metalevel component that required them to make a judgment or decision about the accuracy of their perceptual discrimination judgment. It is important to note that participants always completed the implicit task before the explicit task to avoid transfer of explicit metacognitive strategies from the latter to the former. The structure of the object-level component was identical in each task, but the metalevel component differed in each. Below, we describe first the object-level component that was common to both tasks and then the specific procedures of each task individually.

##### Object-level perceptual discrimination component

Two versions of the object-level component were created and participants completed one version in the gambling task and the other version in the judgment-of-confidence task. In one version (color stimuli version), stimuli comprised pairs of arrays that varied in shade of blue (from very dark to very light blue). On each trial, participants were presented with two arrays, and their task was to select (via mouse click) the array displaying the lightest shade of blue. In the other version (pixel stimuli version), stimuli comprised pairs of arrays that varied in pixel density (from very densely to very sparsely pixelated). On each trial, participants were presented with two arrays, and their task was to select (via mouse click) the array that was most densely pixelated. In each version (color/pixel), each pair varied in terms of how perceptually similar its members were. The more similar the arrays in each pair were, the more difficult it was to discriminate between them. In each version, there was a range of trial difficulty with some trials involving highly similar pairs of arrays and other trials involving highly dissimilar pairs of arrays, along with a wide range in between. Similarity between arrays across trials was determined using a random number generator. For pixel trials, the difference in pixel numbers between the two arrays was generated using a random number generator with requested outputs of 10–80, based on pilot testing to determine average discrimination sensitivity. For color trials, variation in blue color between arrays was determined by varying the luminance of each array, in python RGB color space, and was again generated by a random number generator with requested outputs of 0.001 to 0.5 (again based on pilot testing to determine average discrimination sensitivity). All participants received the same stimuli sets. Task scripts and stimuli parameters are published in an open access GitHub repository (https://github.com/cathgrainger/Explicit-Implicit-JOC-tasks). Importantly, pilot testing was conducted to ensure that the two versions (color/pixel) were of equivalent difficulty and that object-level perceptual discrimination performance was well above chance but well below ceiling on both. Half of the participants in each group were assigned the color stimuli version for the implicit (gambling) task and the pixel stimuli version for the explicit (judgment-of-confidence) task, and half were assigned the pixel stimuli version for the implicit task and the color stimuli version for the explicit task. All stimuli were presented on a 22-in computer screen. We now describe the specific procedure involved in each task.

##### Implicit (gambling) task

**Figure 1 fig1:**
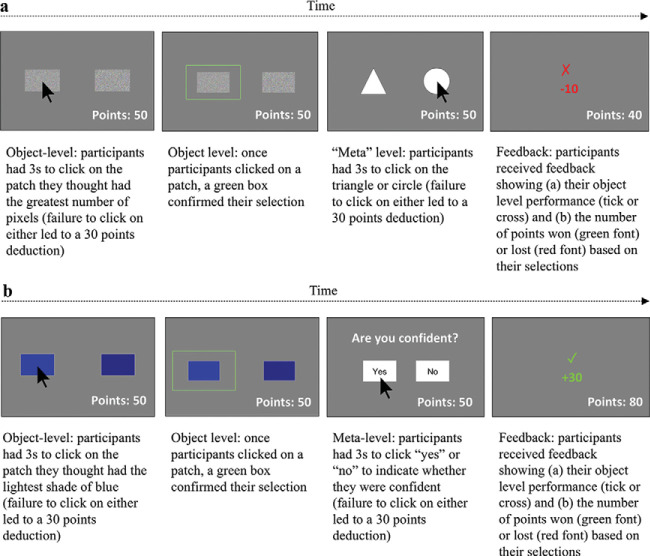
a. Details of the procedure for the implicit (gambling) task. Schematic representation of one trial of the implicit gambling task (pixel version) from Experiment 1. For the object-level component of each trial, participants either completed the color or pixel version. In the pixel version, participants had to click on the patch which had the greatest number of pixels (here the correct answer is the right patch). For the “meta”-level component, participants were presented with a triangle and a circle and asked to click on one of these. After selecting a shape, participants received feedback based on the accuracy (object-level) and which shape they chose (metalevel), as displayed on the far right of the image (in this example, the participant has lost 10 points because they selected the circle [low risk option] having made an incorrect object-level perceptual judgment). Participants had 3 s to make each of their selections, and if the failed to do so for either the object or “meta” level, they received miss feedback. Participants began with 50 points, which was displayed on the bottom right of the screen, and this value updated on each trial based on their feedback, giving participants on ongoing awareness of their score. b. Details of the procedure for the explicit (judgment of confidence) task. Schematic representation of one trial of the explicit judgment of confidence task (color version). For the object-level component, if participants had completed the pixel version of the implicit task, they would complete the color version for the explicit task and vice versa. This time after making their object-level selection, they were asked “Are you confident?” and could select “Yes” or “No.” Participants then received feedback based on their selections at the end of each trial. Participants had 3 s to make each of their selections and if the failed to respond to either the object or meta level decision within this time they received miss feedback. Once again in both tasks, participants began with 50 points, which was displayed on the bottom right of the screen, and this value updated on each trial based on their feedback, giving participants on ongoing awareness of their score.

The main procedure for the implicit task (object- and “meta” components) is illustrated in Figure 1a (note that “meta-” is written in inverted commas here to highlight the fact that, while this phase of the task is assumed by some researchers to require metacognitive monitoring, we did not believe that it necessarily did). This task was modeled closely on methods that have been employed with nonhuman animals in previous research. It was explained to participants that they would be shown a series of pairs of patches (arrays) on a computer screen that either differed in the number of pixels contained in each member of the pair (if they had been assigned to the pixel stimuli version), or in the shade of blue (if they had been assigned to the color stimuli version). Accordingly, they were instructed to select the patch with the greatest number of pixels or the patch with the lightest blue using the computer mouse and were told that they would have 3 s to make each selection. This was the object-level component, described in the preceding section.[Fig-anchor fig1]

The “meta-” level component was subsequently introduced to participants (without using any metacognitive language) in the following way. The experimenter explained that, after each of these perceptual discrimination judgments had been made, a new screen would appear that displayed a square and a triangle and that the participant must select one of these shapes via mouse click. Participants were told that they would start with a balance of 50 points and would win or lose points depending on their responses. They were informed that at the end of the task, they had the chance to win a prize depending on how many points they gained on the task (in fact, at the end of the experiment, all participants received a prize). They were given written and verbal instructions as follows:

a If you click on the patch with most pixels (or lightest blue patch, if completing the color version) and choose the triangle, you will win 30 points;
b If you click on the patch with most pixels (or lightest blue patch) and choose the circle, you will win 10 points;
c If you click on the patch with fewest pixels (or deepest blue patch) and choose the triangle, you will lose 30 points;
d If you click on the patch with fewest pixels (or deepest blue patch) and choose the circle, you will lose 10 points.

As such, the triangle represented the “high risk” symbol (large reward if the perceptual discrimination was correct but large loss if the perceptual discrimination was incorrect), and the circle represented the “low risk” symbol (small reward if the perceptual discrimination was correct, but small loss if the perceptual discrimination was incorrect). It is important to stress, however, that participants never had the rules described to them in these terms, and great care was taken not to use metacognitive language when instructing them.

After the task had been explained and any requests for clarification had been addressed, participants completed 10 practice trials on which their responses did not count toward their final points total. They were asked whether they understood the rules and given a final chance to ask for clarification before beginning the actual task, but no further information about the nature of the task or the study aims was offered. At this point they were asked to complete the 60 experimental trials. The participant’s running total of points was displayed on the bottom left of the computer screen throughout the experiment and updated automatically trial by trial.

At the end of the implicit task, participants’ memory for the response rules was tested by presenting each rule (shown in bullet points *a* to *d* above) and asked them to complete the points value for each response type. The difference in the proportion recalled correctly by participants with ASD (*M* = .88, *SD* = .22) and comparison participants (*M* = .95, *SD* = .13) was statistically small and nonsignificant, *t* = 1.39, *p* = .17, *d* = 0.39, BF^10^ = 0.62 (see the section “statistical analysis” for explanations of the statistical tests used).

Dependent variables for the implicit task were calculated using “Type I” and “Type II” signal detection theory. Type I/Object-level sensitivity was calculated as *d*′ (i.e. participant ability to discriminate between the two perceptual arrays) and reported in RMS (root mean square) units. Object-level response bias (c) was also calculated in RMS units. “Type II” signal detection theory was used to characterize the metacognitive sensitivity of participants’ confidence reports to their correct or incorrect judgments using the meta-*d*′ statistic (see Fleming & Lau, 2014; Maniscalco & Lau, 2012). Meta-*d*′ was fit to confidence rating data using a Maximum Likelihood Estimation (MLE) model, implemented in open access Python code (please see; http://www.columbia.edu/~bsm2105/type2sdt/). Employing a Meta-*d*′ approach allowed for metacognitive sensitivity to be compared with object-level decision accuracy (“Type I” signal detection theory; *d*′) to provide a relative measure of metacognitive efficiency (meta-*d*′*/d*′), controlling for object-level task performance. Metacognitive efficiency is the key variable of interest in the current study because it provides a measure of metacognitive monitoring accuracy that is unbiased by cognitive-/object-level performance. In order to avoid cell counts of 0 interfering with meta-*d*′ model fitting, a correction of 0.25 was added to each stimuli response count, using the correction formula 1/(2*numRatings), as recommended by Maniscalco and Lau (http://www.columbia.edu/~bsm2105/type2sdt/). Finally, the degree of participants’ “risk taking” was calculated as the proportion of times the high-risk triangle symbol was selected (this can also be thought of as type-2 response bias).

##### Explicit (judgment of confidence) task

The main procedure for the explicit condition is illustrated in Figure 1b. The general procedure and points structure were identical to the implicit task. The key difference was that on each trial of the explicit task, after each perceptual discrimination judgment, a new screen appeared containing the question, “Are you confident?” and the words “yes” or “no”, rather than a square and a triangle. Participants were told that, on each trial, if they felt confident they had chosen the most densely pixelated patch (or lightest blue patch, if completing the color stimuli version), they should select the “yes” option via mouse click. In contrast, if they did not feel confident they had chosen the most densely pixelated patch (or lightest blue patch, if completing the color stimuli version), they should select the “no” option via mouse click. Participants were given a set of instructions outlining the consequences of each choice and informed that, at the end of the task, they had the chance to win a prize depending on how many points they gained on the task (in fact, at the end of the experiment, all participants received a prize). Just as for the implicit gambling task, participants were told that they would start with a balance of 50 points and would win or lose points depending on their responses. They were given written and verbal instructions as follows:

e If you click on the patch with most pixels (or lightest blue patch, if completing the color stimuli version) and answer “yes”, you will win 30 points;
f If you click on the patch with most pixels (or lightest blue patch) and answer “no”, you will win 10 points;
g If you click on the patch with fewest pixels (or deepest blue patch) and answer “yes”, you will lose 30 points;
h If you click on the patch with fewest pixels (or deepest blue patch) and answer “no”, you will lose 10 points.

Participants completed 10 practice trials and were then asked whether they understood the rules and were given a final chance to ask for clarification before beginning the experimental task, but no further information about the nature of the task or the study aims was offered. At this point they were asked to complete the main experimental task, comprising 60 trials. The participant’s running total of points was displayed on the bottom left of the computer screen throughout the experiment and updated automatically trial by trial.

At the end of the task, the participant’s memory for the response rules was tested; the experimenter presented the participant with each rule (shown in bullet points e to h above) and asked them to complete the points value for each response type. The difference in the proportion recalled correctly by participants with ASD (*M* = .96, *SD* = .12) and comparison participants (*M* = .97, *SD* = .08) was statistically small and nonsignificant, *t* = 0. 35, *p* = .73, *d* = 0.10, BF^10^ = 0.29.

Explicit task performance was calculated using the same measures as implicit task performance (see above) with four key response measures: (a) object-level sensitivity, calculated as *d*′; (b) object-level response bias, calculated as c; (c) metalevel bias, calculated as the proportion of times the “yes” option was selected when asked “Are you confident?”; and (d) metacognitive efficiency, calculated as a meta *d*′ to *d*′ ratio. As with analysis of the implicit gambling task data, metacognitive efficiency is the key variable of interest in the current paper because it provides a measure of metacognitive monitoring accuracy that is unbiased by object-level performance.

#### Statistical power and analysis

An alpha level of .05 was used to determine statistical significance. Where *t* tests were used, we report Cohen’s *d* values as an index of effect size (≥0.20 = small effect, ≥0.50 = moderate effect; ≥0.80 = large effect; Cohen, 1969). Where ANOVAs were used, we report partial eta squared (η_*p*_^2^) values as an index of effect size (≥.01 = small effect, ≥.06 = moderate effect, ≥.14 = large effect; Cohen, 1969). Where correlations are reported, we use Pearson’s r as an index of effect size (≥.01 = small effect *r* ≥.30 = moderate effect, ≥.50 = large effect), but note that correlations were not a key focus of the current investigation (see General Discussion).

To estimate the necessary sample size to detect the key results, a power calculation was conducted using G*Power3 (Faul, Erdfelder, Lang, & Buchner, 2007). The main aim of Experiment 1 was to establish whether judgment-of-confidence accuracy is significantly diminished in ASD (which we predicted would be the case) and/or whether gambling accuracy is significantly diminished in ASD (which we predicted would not be the case). However, considerations regarding sample size were not straightforward. First, estimating an effect size for group differences in key dependent variables was a challenge. There is little previous research on metacognitive monitoring in ASD and existing studies have used a variety of tasks that are arguably not directly comparable (e.g., Cooper, Plaisted-Grant, Baron-Cohen, & Simons, 2016; Grainger, Williams, & Lind, 2016a, 2016b; Nicholson et al., 2019; Williams, Bergström, & Grainger, 2018; Williams & Happé, 2010; Williams & Happé, 2009; Wilkinson, Best, Minshew, & Strauss, 2010; Wojcik, Allen, Brown, & Souchay, 2011; Wojcik et al., 2014), so basing an effect size on the weighted effect size across previous studies was inappropriate, in our view. Second, we predicted a null effect for the between-groups difference in gambling accuracy, which makes estimating “sufficient power” difficult. Therefore, we adopted two approaches.

First, based on theoretical principles, we estimated that the magnitude of the between-groups difference in judgment-of-confidence accuracy would be equivalent to the magnitude of the between-groups difference in mindreading task performance (*d* = 0.88, according to Yirmiya, Erel, Shaked, & Solomonica-Levi’s [1998] meta-analysis). Twenty-five participants per group provides power of .86, assuming an alpha level of .05 using two-tailed tests (or .96 if using one-tailed tests), which meets Cohen’s (1992) criteria for sufficient power.

Second, we calculated a Bayes factor (BF^10^) for each key analysis. Bayesian analyses supplement null hypothesis significance testing by providing an estimation of the relative strength of a finding for the alternative hypothesis over the null, or vice versa. This allows a more graded interpretation of the data than is possible using *p* values or effect sizes alone (e.g., Dienes, 2014; Rouder, Speckman, Sun, Morey, & Iverson, 2009) and is particularly useful for interpreting null findings because it provides an index of the extent to which data supports the null over the alternative hypothesis (unlike a nonsignificant *p* value, which indicates only that we should not be confident the alternative hypothesis is supported). BF^10^ values can be considered to reflect the probability that the alternative hypothesis is more likely to be true than the null hypothesis. Hence, a BF^10^ of 3 suggests the alternative hypothesis is three times more likely to be true than the null hypothesis. According to Jeffreys’ (1961) criteria, Bayes factors (BF^10^) > 3 provide firm evidence for the alternative hypothesis (with values >10, >30, and >100 providing strong, very strong, and decisive evidence, respectively), and values under 1 provide evidence for the null (with values <0.33 providing firm evidence). Bayesian analyses were conducted using JASP 0.8.1 (JASP team, 2016).

### Results

Table 3 shows object level performance (sensitivity and bias), as well as metalevel performance (M-ratio scores) among participants from each diagnostic group (ASD/NT) on each of the tasks (implicit/explicit) tasks. Meta *d*′ was calculated separately for implicit task performance in the ASD group (*M* = .91, *SD* = 84), and NT group (*M* = 1.03, *SD* = .71), as well as for explicit task performance in the ASD group (*M* = .88, *SD* = .74) and NT group (*M* = 1.36, *SD* = .82). These were then used to calculate metacognitive efficiency as an m-ratio. M-Ratio scores (meta-*d*′/*d*′) were subject to a group (ASD/TD) × task (Implicit/Explicit) mixed ANOVA (see Table 1 for descriptive statistics), which yielded a nonsignificant effect of task, *F*(1, 48) = 0.20, *p* = .66, η_*p*_^2^ = .004, and a nonsignificant effect of group, *F*(1, 48) = 0.49, *p* = .49, η_*p*_^2^ = .01. However, a significant Group × Task interaction was found, *F*(1, 48) = 4.01, *p* = .05, η_*p*_^2^ = .08. Independent samples *t* tests indicated that the M-ratio score was significantly diminished among participants with ASD on the explicit task, but not the implicit task.[Table-anchor tbl3]

Finally, judgment-of-confidence M-ratio score was nonsignificantly associated with implicit gambling M-ratio, *r* = −.001, *p* > .99, or with performance on the RMIE mindreading task, *r* = .06, *p* = .67. However, judgment-of-confidence M-ratio was associated significantly with performance on the Animations mindreading task, when analyzed using one-tailed tests of significance, *r* = .24, *p* < .05. In contrast, implicit gambling M-ratio was nonsignificantly associated with either RMIE or Animations mindreading measures, *r*s <.03, *p*s > .43.

### Summary

In keeping with multiple previous findings, we found metacognitive monitoring accuracy to be significantly diminished among children/adolescents with ASD on an explicit judgment of confidence task. In contrast, “meta-” level performance on an implicit gambling measure was undiminished among participants with ASD. These findings are in line with our predictions that individuals with ASD should have metacognitive impairments and that gambling tasks of the sort used among nonhuman primates do not necessarily measure metacognition, contrary to the claims of many comparative psychologists. Indeed, metalevel performance on the implicit task was not associated significantly with metalevel performance on the explicit task, further suggesting that gambling tasks of this kind do not necessarily measure metacognition. Finally, metalevel performance on the explicit task, but not the implicit task, was associated significantly with mindreading ability (performance on the Animations mindreading task, at least). We will return to address these associations in the General Discussion. In Experiment 2, we examine the key issues using a dual-task design.

## Experiment 2

### Method

#### Participants

160 participants (25 male) with a mean age of 19.35 (*SD* = 2.18) years were recruited from the University of Kent in exchange for course credits as part of their degree. All participants provided informed consent and reported no history of ASD. The study was ethically approved by the University of Kent School of Psychology Research Ethics Committee (Ref: 201715084953804613).

#### Materials and procedures

Each participant completed one of the two tasks used in Experiment 1 (implicit gambling or explicit judgment of confidence) under one of two conditions (single- or dual-task). There were 40 participants in each condition. There were no significant differences between conditions in terms of participant age, *F*(1, 159) = 1.15, *p* = .33, η_*p*_^2^ = .02, or gender, χ^2^ = 2.42, *p* = .49, ϕ = .12. Participants who undertook the gambling or judgment-of-confidence task under single task conditions also completed the mindreading measures (RMIE & Animations) used in Experiment 1 (note that these mindreading measures were not employed among participants who completed the dual-task conditions because the dual-task conditions already taxed mindreading). One participant who completed the explicit dual-task condition was excluded from the results based on being a significant outlier, with an M-ratio score that was more than 5.5 standard deviations from the mean. No other participant scored ≥3SD different from the mean in any condition.

The gambling and judgment of confidence tasks were identical to those used in Experiment 1, except participants in Experiment 2 received real financial incentives, rather that the points for prizes that children/adolescents had won in Experiment 1. Each point in Experiment 1 was equivalent to 1p (pence) in Experiment 2. Hence, in the judgment-of-confidence task, for example, if the participant clicked on the patch with most pixels (or lightest blue patch) and they selected “yes”, they would win 30p (rather than the 30 points that children/adolescents won in Experiment 1). If they chose the patch with most pixels (or lightest blue patch) and they selected “no”, they would win 10p (rather than the 10 points that children won in Experiment 1), and so forth. Participants began with a balance of £2.00 and were told that they would win or lose money based on their responses on each trial. The gambling and judgment-of-confidence payment rules were the same regardless of whether participants completed them under single or dual-task conditions. Participants who completed one of the tasks under dual-task conditions also completed an auditory mindreading task concurrently and received financial incentives for that secondary task also.

For the auditory mindreading task, participants heard via headphones a collection of auditory stimuli taken from the Cambridge Mindreading Voice battery (Golan, Baron-Cohen, & Hill, 2006). These are a series of 50 short phrases, spoken by professional actors and ranging from 2–3 s in length, projecting different emotional and mental states. At the start of a dual task trial, a phrase was presented, followed by two mental state words, the target mental state and a foil. Mental state words were presented in sequence, the first 1 s after the end of the mental state phrase (mental state option 1), and then the other 1 s later (mental state option 2). The order in which targets and foils were presented was counterbalanced across clips. For example, on one trial, participants heard the phrase “That’s not what I was told”, followed by the words “Uneasy” (correct response) and “Provoked” (foil response). In another trial, participants heard the phrase, “That is horrible,” followed by the words “Disturbed” (foil response) and “Appalled” (correct response). The participant’s task was to repeat the word that they judged to reflect the mental state of the speaker of the phrase. They had 3 s to respond on each trial before the next one began. The auditory stimuli were presented throughout the gambling or judgment of confidence tasks (in a fixed order). Participants were informed that for every correct response on the secondary mindreading task, they would win 30p; for every incorrect response, they would lose 30p; and if they failed to respond on a given trial, they would lose 30p. All participants performed above chance (>.50) in each condition on the secondary mindreading task (*M* = .79, *SD* = .14 in the implicit condition and *M* = .77, *SD* = .09 in the explicit condition) and, thus, none was excluded for failure to engage in the secondary task. Explicit feedback, on a trial-by-trial basis on the secondary task was not provided, and participants were told at the end of the task what their reward was for performance on the dual task.

Participants began with a practice task, which was split into three parts. In the first part, they completed 10 trials of the primary gambling or judgment-of-confidence task alone (identical to the practice task completed by participants in the single-task condition). In the second part, they completed five trials of the secondary mindreading task alone. In the final part, they practiced both the primary and secondary tasks together across 10 trials of the primary task.

#### Statistical power and analysis

A power calculation using G*Power3 (Faul et al., 2007) revealed that, to detect a between-condition (single/dual task) difference in judgment-of-confidence accuracy of *d* = 0.88, on 80% of occasions (Cohen, 1992) using two-tailed tests, 22 participants per condition were required. Bayesian analyses were also conducted to enable interpretation of the between-condition difference in gambling accuracy (which we predicted would be null).

### Results

Table 4 shows object level performance (sensitivity and bias), as well as metalevel performance (M-ratio scores) among participants in each condition (single/dual) on each of the tasks (implicit gambling/explicit judgment of confidence) tasks. Meta *d*′ was calculated separately for implicit task performance in the single-task condition (*M* = 1.38, *SD* = 95) and dual-task condition (*M* = 1.33, *SD* = .95), as well as for explicit task performance in the single-task condition (*M* = 1.76, *SD* = 76) and dual-task condition (*M* = 1.13, *SD* = 1.05). These were then used to calculate the key measure of metacognitive efficiency as an M-ratio. M-Ratio scores (meta-*d*′/*d*′) were subject to a condition (single/dual) × task (implicit gambling/explicit judgment of confidence) ANOVA, which yielded a nonsignificant effect of task, *F*(1, 155) = 2.16, *p* = .14, η_*p*_^2^ = .01. The effect of condition was significant, *F*(1, 155) = 8.27, *p* = .005, η_*p*_^2^ = .05, as was the Task × Condition interaction, *F*(1, 155) = 4.33, *p* = .03, η_*p*_^2^ = .03. Independent samples *t* tests indicated that, on the explicit judgment of confidence task, the M-ratio score was significantly lower in the dual-task condition than in the single-task condition. On the implicit gambling task, however, the M-ratio score did not differ significantly across conditions (see Table 4).[Table-anchor tbl4]

Correlation analyses revealed that judgment-of-confidence M-ratio score was nonsignificantly associated with performance on the RMIE mindreading task, *r* = −.15, *p* = .34, but was associated significantly with performance on the Animations mindreading task, when analyzed using one-tailed tests of significance, *r* = .26, *p* = .05. In contrast, implicit gambling M-ratio was nonsignificantly associated with either RMIE or Animations mindreading measures, *r*s < −.25, ps > .12.

### Summary

In line with predictions, concurrent completion of a secondary mindreading task impaired metacognitive monitoring accuracy among neurotypical adults on a judgment of confidence task. In contrast, concurrent completion of this secondary mindreading task did not diminish “meta”-level performance on an implicit gambling measure of the sort claimed to assess metacognitive monitoring among nonhuman primates. These results mirror and complement those found in Experiment 1, in which a group of people with mindreading impairments (i.e. those with ASD) showed diminished metalevel performance on the explicit judgment of confidence task, but not the implicit gambling task.

It is clear from the results in Experiment 2 (as well as Experiment 1) that implicit gambling accuracy cannot rely on mindreading resources in the same way that explicit judgment-of-confidence accuracy appears to, given that only the latter was detrimentally affected by concurrent completion of a secondary task that tapped mindreading. However, there is a possibility that the detrimental effect of secondary-task completion on judgment-of-confidence accuracy in Experiment 2 was not because the tasks shared a common metarepresentational processing resource. Rather, it could be that concurrent completion of any secondary task, regardless of the processing resources tapped by the secondary task, would detrimentally affect judgment-of-confidence accuracy. In other words, it might be that metacognition is disrupted by imposition of any additional cognitive load (a general dual-task effect), rather than by imposition of additional metarepresentational load specifically (a specific dual-task effect). To address this, we devised two additional dual-task conditions and had new groups of neurotypical participants complete the judgment-of-confidence task under one of these conditions (*n* = 31 per condition). We then compared judgment-of-confidence accuracy in the single- and standard dual-task conditions (reported above) with judgment-of-confidence accuracy in each of these additional dual-task conditions.

In one condition (gender, sentence forward condition), participants listened to the same auditory stimuli (from the Cambridge Mindreading Voice battery) that participants in the standard dual-task condition completed in Experiment 2, but judged the gender of the speaker (male/female) rather than the thought/emotion the speaker was experiencing. In this condition, even though the task was to judge the gender of the speaker, we thought it was possible that the thought/emotion the speaker was experiencing could be processed automatically. If the thought/emotion of the speaker was processed automatically, even though it was not the goal of the task, then performing this secondary task concurrently with the explicit judgment of confidence task might reduce metacognitive efficiency (because, according to our predictions, both tasks depend on mindreading). Therefore, we designed a second control condition to rule out the possibility of automatic activation of the mindreading system during task completion. In this second control condition (gender, sentence backward), the stimuli from the Cambridge Mindreading Voice battery task were rerecorded by adult males and females who used neutral intonation. However, it was not only the intonation of the voices in the Cambridge Mindreading Voice battery task that indicated the thoughts/emotions experienced by the speaker, but also the content of the sentences themselves. Therefore, to rule out automatic inference of mental states in this gender, backward condition, the sentences were spoken in reverse (e.g., “I’ve been waiting so long for this moment” became “moment this for long so waiting been I’ve”). Again, participants were tasked with judging the gender of the speaker, but we reasoned that mindreading would be (near-) impossible in this condition.

## Experiment 3

### Method

#### Participants

Thirty-one participants (4 male) completed the judgment of confidence task alongside the gender, forward sentences dual-task and 31 participants (10 male) completed the judgment of confidence task alongside the gender, backward sentences dual-task. The average age of participants who completed the gender forward sentences dual-task condition was 23.48 years (*SD* = 5.23). The average age of participants who completed the gender backward sentences dual-task condition was 23.10 years (*SD* = 6.64). All participants were recruited from the University of Kent, in exchange for course credits as part of their degree or payment, and from the local community. No participant had a history of ASD, according to self-report. All participants provided informed consent, and the study was ethically approved by the University of Kent, School of Psychology Research Ethics Committee, 201715084953804613.

#### Materials and procedures

In Experiment 3, participants completed the same judgment of confidence task used in Experiment 2 but concurrently with one of two new secondary tasks. The first new task was a “gender, forward sentences” secondary task was identical to the secondary task used in Experiment 2 except in one respect. In this new secondary task, after the presentation of each sentence from the Cambridge Mindreading Voice Battery (Golan et al., 2006), participants heard the words “male” and “female” and had to report verbally which sex the speaker of the sentence had been. Of the 40 stimuli used in the experiment, 24 were spoken by a male actor and 16 were spoken by a female actor. The payment rules for this secondary task were the same as for the secondary task in Experiment 2; for each correct gender judgment, participants won 30p, for each incorrect judgment participants lost 30p, and if they failed to respond they lost 30p. All other procedures, including the judgment-of-confidence task, practice procedure, and payment rules, were identical to those outlined in Experiment 2. One participant was excluded from the results based on being a significant outlier, with an M-ratio score that was more than 5.5 standard deviations different from the mean. No other participant scored ≥3 SD different from the mean in any condition.

The second new task was a “gender, backward sentences” secondary task. For this new task, the auditory stimuli from the Cambridge Mindreading Voice Battery (Golan et al., 2006) was rerecorded by three female and three male actors using a neutral voice. As the actors removed any intonation, emphasis, and emotion from their voice, any mental states automatically associated with the speech would be reduced. In order to also rule out any possibility of automatic mindreading, actors spoke each sentence backward in order to minimize emotional content of the sentences. So, for example, “He never helps at home” was recorded as “home at helps never he.” Participants heard these new sentences and after each heard the options “male” and “female.” Their task was to identify the gender of the speaker for each sentence by verbally reporting one of the two genders. All other aspects of the task were identical to those employed in the gender, forward dual-task. All participants performed above chance (>.50) in each of the secondary tasks and, thus, none was excluded for failure to engage in the secondary task.

### Results

Meta *d*′ was calculated for performance in the gender forward dual-task condition (*M* = 1.14, *SD* = .77) and the gender backward dual-task condition (*M* = 1.43, *SD* = .83). Means and standard deviations for each dependent variable for the judgment-of-confidence task in each of the supplementary dual-task conditions are reported in Table 5. Independent *t* tests revealed no significant effects between conditions in any of the variables. Next, we compared judgment-of-confidence performance in each of these control conditions with judgment-of-confidence performance in each of the single- and dual-task conditions completed by participants in Experiment 2. An initial series of ANOVAs revealed a significant effect of condition (single-/dual-mindreading/dual-gender forward sentences/dual-gender-backward) on M-ratio, *F*(3, 136) = 3.94, *p* = .01, η_*p*_^2^ = .08, but not object-level sensitivity, *F*(3, 136) = 0.32, *p* = .81, η_*p*_^2^ = .007, or object-level bias, *F*(3, 136) = 1.89, *p* = .13, η_*p*_^2^ = .04. Independent-samples *t* tests revealed that M-ratio in the single task condition (from Experiment 2) was nonsignificantly different from M-ratio in either the dual-gender-forward sentences condition, *t*(68) = 1.15, *p* = .25, *d* = 0.28, BF^10^ = 0.44, or the dual-gender-backward sentences condition, *t*(69) = 0.39, *p* = .70, *d* = 0.09, BF^10^ = 0.26. In other words, metacognitive monitoring accuracy was nonsignificantly affected by completion of either of the control dual-task conditions. In contrast, M-ratio, in the dual-mindreading task condition (from Experiment 2) was significantly lower than in either the dual-gender-forward sentences condition, *t*(68) = 2.03, *p* = .05, *d* = 0.49, BF^10^ = 1.41, or the dual-gender-backward sentences condition, *t*(68) = 2.56, *p* = .01, *d* = 0.62, BF^10^ = 3.81.[Table-anchor tbl5]

### Summary

In Experiment 3, we designed two control dual-task conditions, a gender, forward sentences condition and a gender, backward sentences condition. Participants completed the explicit judgment-of-confidence task under one of these two control conditions. The aim was to show that completing an explicit metacognitive task alongside a secondary task that did not require mindreading would not have a significant detrimental effect on metacognitive accuracy. This is what we found. Metacognitive monitoring accuracy on the primary judgment-of-confidence task was nonsignificantly affected by completion of either of the control dual-task conditions (reported in Experiment 3), relative to performance in the single-task condition (reported in Experiment 2). Indeed, the only dual-task condition to significantly negatively influence judgment-of-confidence accuracy was the mindreading dual-task condition reported in Experiment 2. Judgment-of-confidence accuracy was significantly lower in the mindreading dual-task condition than in any of the other three conditions (single-task, dual-task forward-sentences, dual-task backward-sentences). This suggests that it is mindreading specifically that is linked to metacognitive monitoring and that taking up the resources required for the former has a negative effect on the latter, in keeping with our predictions.

## General Discussion

Across three experiments, we found consistent evidence in support of our key hypotheses. To elaborate, with regard to our first question about whether metacognition and mindreading share the same metarepresentational resources (the one-system view), we found that judgment-of-confidence accuracy was significantly diminished in ASD, indicating that metacognition is impaired in this disorder. Thus, contrary to the claims of some that metacognition is unimpaired whereas mindreading is diminished in ASD, we found no evidence of a dissociation between metacognition and mindreading among participants with ASD. This finding is in keeping with the prediction made by one-system views of the relation between metacognition and mindreading and complements data obtained using other methods (Nicholson et al., 2019).

Of course, it is possible that ASD involves a “double hit” of impairments that disrupt both metacognition and mindreading. Such a finding alone would be clinically important but wouldn’t necessarily count against two-systems or metacognition-is-prior views. However, if the mindreading and metacognitive impairments in ASD were the result of a double hit of independent impairments, rather than as a result of a single metarepresentational faculty that processes mental states in others and self, then there is no reason to suppose that taking up mindreading resources in a dual-task paradigm would disrupt metacognition among neurotypical individuals. Yet, in Experiment 2, we found judgment-of-confidence accuracy was selectively impaired by concurrent performance on a secondary mindreading task. This suggests that the primary metacognition and secondary mindreading tasks were competing for metarepresentational resources in a way that is predicted only by one-system theories.

However, there is also the theoretical possibility—mentioned, but set aside, in the introduction—of a version of metacognition-is-prior view that claims a damaged metacognitive system is the primary cause of mindreading deficits in ASD. While to the best of our knowledge no one has proposed such a view, it is a possible view, and nothing in the experiments conducted here rules it out.

It likewise remains possible that the reduced metacognitive performance among participants with ASD in the explicit (but not implicit) metacognition task was caused by something other than reduced mindreading. The implicit and explicit tasks also differ in the demands they make on linguistic ability, for example. While we cannot exclude the possibility that there is *some* factor other than mindreading that explains the differential performance in ASD, it is unlikely to be a difference in linguistic abilities, since these were closely matched with the control group.

Turning now to Experiments 2 and 3, it is important to highlight that none of the task variables other than metacognitive accuracy was affected by concurrent performance on the secondary mindreading task. Hence, it is not the case that merely performing a secondary task disrupts primary task performance *per se.* Rather, it was metacognitive accuracy that was selectively diminished by concurrent mindreading (and continued to be diminished even after the influence of all other task variables was controlled). Finally, the detrimental effect on judgment-of-confidence metacognitive accuracy of concurrent completion of a secondary task appeared to be specific to when the secondary task involved mindreading (in Experiment 2). When the judgment of confidence task was completed alongside a dual-task that did not demand mindreading (in Experiment 3), then metacognitive accuracy was equivalent to that in the single-task condition reported above. Thus, it was not the case that concurrent completion of any secondary task disrupted metacognitive accuracy, but rather that metacognitive accuracy was detrimentally affected when metarepresentational processing resources were taxed by the secondary task. While this result is consistent with our one-system view, taken on its own it is also consistent with a metacognition-is-prior account.

We were unable to control for all possible differences between the dual-tasks employed in Experiments 2 and 3, of course. It may be that judging emotion from speech in order to make binary selections among candidate emotion words, where the candidates vary from trial to trial (Experiment 2), is inherently more difficult than making repeated binary judgments of gender (Experiment 3). Perhaps the former task is more demanding of attention and/or working memory. If that were the case, then it may be that this additional load, rather than a specific drain on mindreading resources, interfered with the explicit judgment-of-confidence task. Future studies might explore this possibility further.

In keeping with our predictions, metalevel performance on the explicit judgment of confidence task was associated significantly (using one-tailed tests of significance) with performance on the Animations measure of mindreading in both Experiments 1 and 2, which replicates the finding of Nicholson et al. (2019) who observed a similar sized association between judgment of confidence accuracy and Animations task performance (*r* = .35 in their study).

One issue to address, however, is the fact that RMIE mindreading task performance was not associated significantly with judgment-of-confidence accuracy in either Experiment 1 or 2, contrary to our predictions and to the findings of Williams et al. (2018) and Nicholson et al. (2019). We do not have a concrete explanation for the nonsignificant association between judgment of confidence accuracy and mindreading in the current study. However, we note that an investigation into the correlations between mindreading and metacognition was not a key aim of the current study, and we were aware that the study was not sufficiently powered to detect the association predicted by one-system theorists (note that replication studies likely require between 2.5 and 3.5 times the sample size of the original studies; e.g., Simonsohn, 2015, 2016). There is now a clear pattern emerging across studies that indicate a consistently modest, but reliable, association between metacognition and mindreading. Significant associations between judgment of confidence accuracy and RMIE task performance have been observed in independent samples by Williams et al. (2018, *r* = .25), Nicholson et al. (2019, *r* = .26), and Carpenter & Williams (under review, *r* = .26). To detect a correlation of .26 on 80% of occasions, a sample of 90 participants is required even when using one-tailed tests. Therefore, we were aware that our samples of *N* = 50 in Experiment 1 and *N* = 40 in Experiment 2 were not sufficiently powered to detect even predicted associations, which we acknowledge as a limitation. Nonetheless, it is reassuring that the significant associations we observed between judgment of confidence accuracy and Animations task performance was of an almost-identical magnitude in both Experiments 1 (*r* = .24) and 2 (*r* = .26) as observed in the few previous studies that have explored the relation between mindreading and metacognition.

In sum, we found converging evidence from three experiments that when mindreading resources are diminished (either clinically in ASD or artificially in neurotypical people when performing a dual task), so too are metacognitive resources. The overall pattern of these findings would not be predicted by either two-systems or metacognition-is-prior theorists, nor can it be explained straightforwardly by either of those theories. Rather, the results support one-system views of the relation between mindreading and metacognition.

Turning to our second question, regarding whether implicit gambling tasks of the sort that have been claimed to measure metacognition in nonhuman primates necessarily require metarepresentation, we also found consistent evidence for our predictions from findings in Experiments 1 and 2. In Experiment 1, our sample of ASD participants showed impairments in metarepresenting self (judgment-of-confidence accuracy) and others (Animations), yet still performed equivalently to the neurotypical comparison group in terms of gambling accuracy. These findings suggest that gambling tasks of the sort used among nonhuman primates do not necessarily require metarepresentation (nor do they seem to involve such metarepresentation in humans who undertake them), but can be achieved using first-order risk-based affective appraisals of likely success.

Again, converging results were observed in Experiment 2. Whereas judgment-of-confidence accuracy was consistently and selectively impoverished by the imposition of a secondary mindreading task, gambling accuracy (despite having an almost identical task structure to the judgment-of-confidence task) was not. The between-condition difference in gambling accuracy was small, nonsignificant, and associated with a Bayes factor that supported the null hypothesis. This shows that, even when metarepresentational resources are taxed (by the secondary mindreading task), gambling is as accurate as when metarepresentational resources are not taxed at all (in the single-task condition).

In addition, across both Experiments 1 and 2, performance on the implicit and explicit tasks was uncorrelated. This result speaks against the view that implicit gambling tasks of the sort used to measure metacognitive processes in monkeys are actually doing so. For even though the implicit and explicit tasks differ along a number of dimensions, if both tap into metacognitive abilities, then performance in each should be significantly correlated with the other. This finding complements the finding by Nicholson et al. (2019) that implicit and explicit performance on another of the tasks used to measure metacognition in monkeys—namely, so-called “uncertainty monitoring” tasks—is likewise uncorrelated. Specifically, in Nicholson et al. (2019), a group of adults with ASD and a matched control group of neurotypical adults completed a classic explicit metacognitive task, similar to the explicit task used in the current study, as well an implicit (“strategic opt-out”) measure that was different to the gambling paradigm used in the current study. The implicit task involved making perceptual discriminations, with success resulting in financial reward and failure resulting in financial penalty. The crucial feature of this paradigm is that participants could choose to opt-out of any given trial and avoid penalty or reward on that trial. This kind of paradigm is used frequently among nonhuman primates, with adaptive use of the opt-out option on difficult trials taken to indicate that the participant metarepresents their own state of uncertainty when faced with a challenging trial and behaves accordingly. Yet, Nicholson et al. (2019) found that participants with ASD made adaptive use of the opt-out option to the same extent as neurotypical participants, despite showing significant impairments in explicit metacognitive accuracy and mindreading accuracy (with performance on the latter two tasks correlated significantly, despite neither being correlated with strategic opting out). Just as in the current study, therefore, Nicholson et al. observed a dissociation between performance on classic metacognitive tasks and tasks of the sort that are claimed to reveal metacognition in nonhuman primates. This calls into question whether such implicit tasks really require metarepresentation of one’s own internal states for successful performance. It remains possible, however, that metacognitive abilities fractionate along a number of different dimensions, with one subset of abilities being accessed by our implicit tasks, and another subset utilized in the explicit tasks. This, too, could explain the lack of correlation.

Even if it were true that humans fail to metarepresent their own states when they gamble accurately in our implicit tasks, it may be that nonhuman primates nevertheless do so. However, our results suggest that one should use caution when interpreting the gambling performance of nonhumans. If humans, a species we know to be capable of accurate metacognition, do not need to metarepresent their own states to gamble accurately, then we should arguably be skeptical of claims that nonhumans do need to metarepresent their states on such tasks. Moreover, if implicit tasks of the sort successfully employed with monkeys are not genuinely metacognitive in nature, then one important strand of support for metacognition-is-prior accounts is undercut.

In sum, the current study has significant implications for both theory development and clinical practice. Taken together, our findings are in keeping with only one of the theories considered, which is striking given that several predictions overlap across theories, highlighting how challenging it is to distinguish them empirically. The findings suggest, on the one hand, that implicit gambling paradigms of the sort used to test “metacognition” in nonhuman animals do not, in fact, measure awareness of one’s own mental states (not in humans, at any rate). On the other hand, the results suggest that accuracy of explicit metacognitive judgments about one’s own mental states depends to a significant extent on mindreading ability. As a result, people with ASD, who have established mindreading difficulties, also show significantly weaker performance on explicit metacognitive tasks (but not implicit gambling tasks).

This work had its roots in two suggestions made by Carruthers (2009, 2011) on theoretical grounds. The first was that the same faculty underlies both mindreading of others and metacognition of self. This view was shared by Williams (e.g., 2010; see also, Williams, Lind, & Happé’s [2009] commentary on Carruthers, 2009), and endorsed by him as an explanation for key difficulties in both other- and self-awareness experienced by many people with ASD. The second suggestion was that so-called nonverbal “metacognitive” tasks employed with nonhuman animals are not genuinely metacognitive in nature. This led to discussions about how best to test these theoretical predictions, resulting in Nicholson et al. (2019) and the present study. Future work may extend the framework to employ other methods or to test other types of alleged implicit metacognition.

## Figures and Tables

**Table 1 tbl1:** Predictions Following From Each Theory

Theory	Prediction				
	ASD implicit task performance is impaired	ASD explicit task performance is impaired	Implicit task correlates with explicit task	Secondary mindreading task selectively affects explicit task only	Secondary mindreading task affects implicit and explicit tasks
Two-systems & implicit tasks are metarepresentational	**No:** because metacognition is not impaired and implicit task does not require mindreading system	**No:** because metacognition is not impaired and explicit task would not require mindreading system	**Yes:** because both implicit and explicit tasks should rely on the same metacognitive system	**No:** because mindreading is distinct from metacognition, leaving both tasks unaffected	**No:** because mindreading is distinct from metacognition, leaving both tasks unaffected
Metacognition-is-prior & implicit tasks are metarepresentational^a^	**No:** because metacognition is not predicted to be impaired and implicit task would not require mindreading system	**No:** because metacognition is not predicted to be impaired and explicit task would not require mindreading system	**Yes:** because both implicit and explicit tasks should rely on the same metacognitive system	**No:** because mindreading depends on metacognition, so both tasks are affected by interference with mindreading	**Yes:** because mindreading depends on metacognition, so both tasks are affected by interference with mindreading
One-system & implicit tasks are metarepresentational	**Yes:** because with one system, metarepresentation in general is impaired in ASD	**Yes:** because with one system, metarepresentation in general is impaired in ASD	**Yes:** because both implicit and explicit tasks should rely on the same metacognitive system	**No:** because both implicit and explicit tasks require the same metarepresentational system as mindreading	**Yes:** because both implicit and explicit tasks require the same metarepresentational system as mindreading
Two-systems & implicit tasks are not metarepresentational	**No:** because damage to mindreading should not lead to deficits in a first-order task	**No:** because damage to mindreading leaves metacognitive system intact	**No:** because implicit tasks are not metarepresentational, whereas explicit tasks are	**No:** because mindreading is distinct from metacognition and is not required for a first-order task, so both tasks unaffected	**No:** because mindreading is distinct from metacognition and is not required for a first-order task, so both tasks unaffected
Metacognition-is-prior & implicit tasks are not metarepresentational^a^	**No:** because damage to mindreading should not lead to deficits in a first-order task	**No:** because metacognition is not predicted to be impaired and explicit task would not require mindreading system	**No:** because implicit tasks are not metarepresentational, whereas explicit tasks are	**Yes:** because mindreading depends on metacognition and only the explicit task is metacognitive	**No:** because mindreading depends on metacognition and only the explicit task is metacognitive
One-system & implicit tasks are not metarepresentational	**No:** because damage to mindreading should not lead to deficits in a first-order task	**Yes:** because explicit task shares metarepresentational system with mindreading which is impaired in ASD	**No:** because implicit tasks are not metarepresentational, whereas explicit tasks are	**Yes:** because only the explicit task involves the mindreading system, so only the explicit task is affected	**No:** because only the explicit task involves the mindreading system, so only the explicit task is affected
*Note*. ASD = autism spectrum disorder.					
^a^ The metacognition-is-prior view represented here is the version actually defended by theorists in the literature, according to which metacognition should not be damaged in ASD. Another possible version of metacognition-is-prior view, according to which metacognitive deficits lie at the base of the well-known mindreading difficulties in ASD, is not addressed in these experiments or in our discussion (except in passing).					

**Table 2 tbl2:** Experiment 1 Participant Characteristics and Group Matching Statistics

Variable	ASD (*n* = 25)	NT (*n* = 25)	*t*	*p*	*d*
Age	12.71 (1.52)	13.17 (1.54)	1.07	.29	0.30
VIQ	105.92 (9.90)	109.20 (10.82)	1.12	.27	0.32
PIQ	109.36 (13.04)	113.56 (13.91)	1.10	.28	0.31
SRS T-score	84.75 (9.45)	45.88 (10.47)	13.62	<.001	3.90
RMIE proportion correct^a^	.69 (.08)	.73 (10)	1.42	.08	0.41
Animations proportion correct	.46 (.24)	.70 (.18)	4.01	<.001	1.14
*Note*. VIQ = verbal IQ; PIQ = performance IQ; SRS = Social Responsiveness Scale; RMIE = Reading the Mind in the Eyes; ASD = autism spectrum disorder; NT = neurotypical.					
^a^ One participant with ASD became distressed when completing this task, so data was collected from only 24 participants.					

**Table 3 tbl3:** Object-Level and Meta-Level Performance on the Gambling and Judgement of Confidence Task, in Autistic and Neurotypical Participants (Experiment 1)

Task	Variable	Group		*t*	*p*	*d*	BF^10^
		ASD	NT				
Gambling	Object-level: Sensitivity (*d*′)	0.99 (0.54)	1.00 (0.59)	0.11	.92	0.03	0.29
	Object-level: Criterion/bias (*c*)	−.32 (.61)	−.07 (.57)	1.47	.15		
	Meta-level: M-ratio	1.04 (1.21)	.60 (2.66)	0.76	.45	0.45	0.36
Judgement of confidence	Object-level: Sensitivity (*d*′)	1.00 (0.60)	1.02 (0.60)	0.11	0.91	0.03	0.29
	Object-level: Criterion/bias (*c*)	−.22 (.33)	−.13 (.57)	.65	.52		
	Meta-level: M-ratio	.51 (1.74)	1.45 (0.99)	2.37	.01	0.67	5.21
*Note*. ASD = autism spectrum disorder; NT = neurotypical; BF = Bayes factor.							

**Table 4 tbl4:** Gambling and Judgement of Confidence Task Performance in the Single and Dual Task Condition (Experiment 2)

Task	Variable	Condition		*t*	*p*	*d*	BF^10^
		Single-task	Dual-task				
Gambling	Object-level: Sensitivity (*d*′)	1.21 (0.68)	1.23 (0.50)	0.16	.87	0.04	0.23
	Object-level: Criterion/bias (*c*)	.02 (.51)	.06 (.59)	0.32	.75	0.07	0.24
	Meta-level: M-ratio	1.33 (1.21)	1.08 (0.87)	1.08	.28	0.24	0.39
Judgement of confidence	Object-level: Sensitivity (*d*′)	1.31 (0.59)	1.27 (.74)	0.25	.80	0.06	0.24
	Object-level: Criterion/bias (*c*)	−.31 (.51)	.07 (1.15)	1.94	.06	0.43	1.17
	Meta-level: M-ratio	1.52 (2.20)	0.02 (3.98)	2.50	.01	0.47	3.34
*Note*. BF = Bayes factor.							

**Table 5 tbl5:** Mean (SD) Performance on Each Variable of the Judgement-of-Confidence Task in Each of the Dual-Task Conditions in Experiment 3

Variable	Condition		*t*	*p*	*d*	BF^10^
	Dual, Gender forwards	Dual, Gender backwards				
Object-level: Sensitivity (*d*′)	1.23 (0.68	1.16 (0.53)	0.40	.69	0.10	0.28
Object-level: Criterion/bias (*c*)	−.24 (.48)	−.44 (1.37)	0.74	.46	0.19	0.33
Meta-level: M-ratio	1.02 (1.06)	1.73 (2.36)	1.52	.14	0.39	0.68
*Note*. BF = Bayes factor.
